# On the reactive states of astrocytes in prion diseases

**DOI:** 10.1080/19336896.2021.1930852

**Published:** 2021-05-31

**Authors:** Ilia V. Baskakov

**Affiliations:** Department of Anatomy and Neurobiology, And Center for Biomedical Engineering and Technology, University of Maryland School of Medicine, Baltimore, MD, USA

**Keywords:** Prion, prion diseases, reactive astrocytes, reactive microglia

## Abstract

Transformation of astrocytes into reactive states is considered one of the major pathological hallmarks of prion and other neurodegenerative diseases. Recent years witnessed a growing appreciation of the view that reactive astrocytes are intimately involved in chronic neurodegeneration; however, little is known about their role in disease pathogenesis. The current article reviews the progress of the last few years and critically discusses controversial questions of whether reactive astrocytes associated with prion diseases are neurotoxic or neuroprotective and whether bidirectional A1–A2 model is applicable for describing polarization of astrocytes. Moreover, other important topics, including reversibility of a transition to a reactive state, along with the role of microglia and other stimuli in triggering astrocyte activation are reviewed. Defining the role of reactive astrocytes in pathogenesis of neurodegenerative diseases will open unrealized opportunities for developing new therapeutic approaches against prion and other neurodegenerative diseases.

## Unsettled questions of astrocyte biology

Astrocytes are responsible for a number of homoeostatic functions required for proper functioning of CNS [1–3]. Under chronic neurodegeneration associated with prion and other neurodegenerative diseases, astrocytes undergo significant transcriptional, morphological and functional transformation resulting in reactive phenotypes [4,5]. Recent years witnessed a growing appreciation of the view that reactive astrocytes are intimately involved in chronic neurodegeneration [6–9]. However, the precise role of reactive astrocytes in disease pathogenesis remains highly controversial (reviewed in [6,10]). A number of important questions remain unsettled. The extent to which normal homoeostatic functions are altered in the reactive states is unknown. It is not clear whether polarization into reactive state produces the net neurotoxic or neuroprotective outcome [6–8]. Another controversial topic is whether microglia trigger astrocyte activation and dictate their reactive phenotype. However, among the most important questions is whether the polarization of astrocytes into reactive states represents a downstream response to altered brain homoeostasis or, on the contrary, drives prion pathogenesis. Finally, it is also unclear whether the transformation of astrocytes into reactive states is fully reversible.

## Is A1–A2 polarization model applicable to prion diseases?

**Figure 1.**
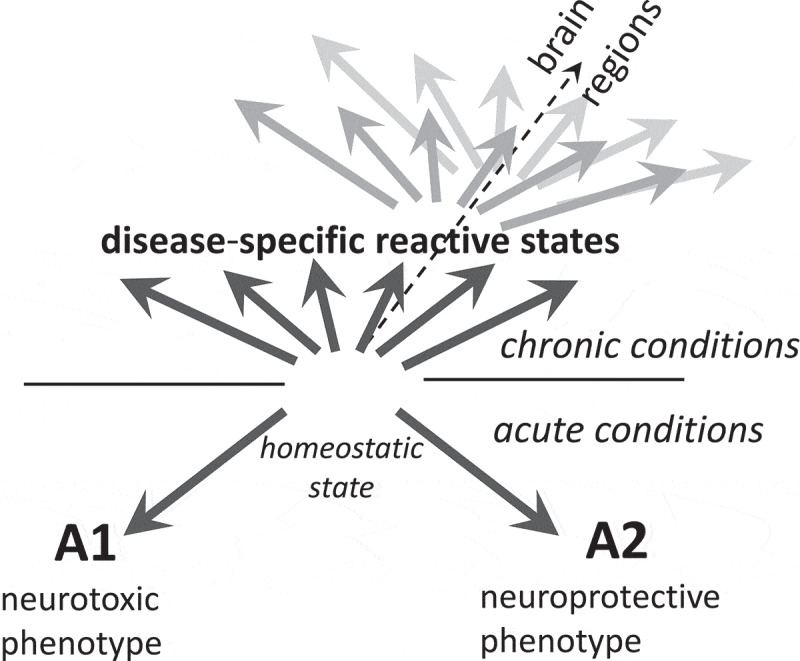
The diagram illustrating that in neurodegenerative diseases, instead of polarization according to the bidirectional A1-A2 or neurotoxic-neuroprotective model, reactive astrocytes adopt multiple, disease-specific states dictated in part by a nature of an insult. Within individual diseases, reactive states might vary across brain regions at any given time point of the disease (represented by dark and light grey arrows)

According to the hypothesis introduced by Barres and co-authors, astrocytes can polarize into well-defined neurotoxic (A1) or neuroprotective (A2) reactive states, which exhibit distinct transcriptional profile and opposing effects on neuronal survival [11,12]. The hypothesis proposing alternative A1 and A2 reactive states was developed using animals treated with LPS or subjected to ischaemic stroke [11], conditions that do not induce long-term chronic effects (Figure 1). The question whether bidirectional polarization model is applicable to chronic neurodegenerative illnesses is highly controversial [10]. Moreover, assessing polarization phenotypes based on animal models is tricky, because most neurodegenerative diseases rely on genetically modified animals that might not faithfully reproduce all aspects of chronic neuroinflammation or neurodegeneration of human diseases [13–15]. Wild type on inbreed animals infected with prions of natural or synthetic origin develops bona fide prion disease [16–20]. As judged from several independent transcriptome studies, in prion-infected animals, astrocytes do not follow the A1–A2 polarization model [21–26]. Instead, upregulation of a mixture of A1-, A2- and pan-reactive markers was observed [21–26]. At present, it is not clear, whether the mixed A1/A2 profile, which was observed in bulk tissues, arise as a result of the actual mixture of A1 and A2 astrocytes, existence of multiple activation states, co-expression A1- and A2-specific markers within individual cells, or all of the above. Application of single cell transcriptome approach should answer the questions regarding diversity of the reactive phenotypes associated with prion diseases along with identifying molecular sub-types not distinguishable from the analyses of bulk tissues. Up to date, very similar profiles of A1-, A2- and pan-reactive markers were found regardless of a brain region or prion strain [21,26], suggesting that in prion diseases, astrocyte adopt relatively uniform reactive state, which might be different from the reactive states in other chronic illnesses. Moreover, the reactive phenotype of astrocytes associated with prion disease was universal across strains with different cell tropism, that is, regardless of whether prion strains prefer to propagate in neurons or astrocytes [26]. Notably, echoing findings in the prion field, a panel of experts in astrocyte biology, recommended moving beyond the A1–A2 labels in describing phenotypes of astrocytes in acute injuries and chronic illnesses of CNS [10].

## Is reactive phenotype associated with prion disease neurotoxic or neuroprotective?

Do reactive astrocytes contribute to diseases pathogenesis? Considering that a mixture of A1- and A2-reactive markers were upregulated in prion diseases, while neither markers being liked to homoeostatic or toxic functions of astrocytes, the bidirectional A1-A2 concept does not provide a cue whether reactive astrocytes are expected to produce neuroprotective or neurotoxic effects. PrP^Sc^ strains that target astrocytes often have shorter incubation times to the disease in comparison with the strains that colocalize with neurons [22,27]. Moreover, transmissible PrP states that are characterized by very mild reactive astrogliosis were shown to propagate in CNS silently, without causing clinical signs of the diseases despite substantial synaptic immunoreactivity [28]. These results brings up a possibility that a causative link between astrocyte response to transmissible PrP states and disease pathogenesis exists.

**Figure 2.**
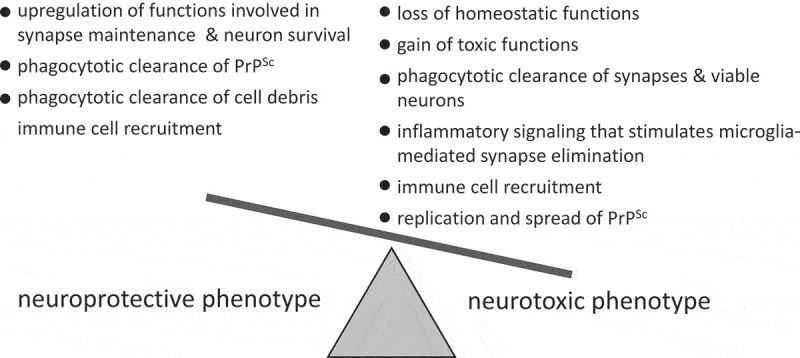
Schematic diagram illustrating that dysregulation of multiple neuroprotective and neurotoxic mechanisms might contribute to defining a net outcome of reactive astrocyte phenotype

Several possible mechanisms, including upregulation of functions involved in synapse maintenance and neuronal survival, phagocytic clearance of PrP^Sc^ and cell debris or, in opposite, spread of PrP^Sc^, elimination of synapses along with viable neurons, loss of homoeostatic functions, gain of toxic functions, inflammatory signalling that stimulates microglia-mediated synapse elimination, recruitment of immune cells and others mechanisms, should be examined to determine the net impact of reactive astrocytes (Figure 2).

Recent studies suggested that reactive astrocytes associated with prion diseases are neurotoxic [29,30]. Using primary astrocyte cultures isolated from prion-infected animals, our recent work revealed that in the reactive states, astrocytes exbibit profound neurotoxic effects mediated via astrocyte-conditioned media and also manifested directly in co-cultures with primary neurons [29]. As tested in co-cultures with neurons, homoeostatic functions responsible for neuronal growth, spine development and synapse maturation were impaired in reactive astrocytes isolated from the prion-infected animals [29]. The media conditioned by the reactive astrocytes too had deleterious effects on primary neurons, including reduction in density and size of the dendritic spines, disintegration of synapses, reduced expression of pre- and post-synaptic proteins along with a decrease in viability of neurons [29]. Genes involved in formation, maturation and stability of synapses and dendritic spine were downregulated in astrocytes derived from prion-infected animals [29]. These results argue that in the reactive state associated with prion disease, the net effect of astrocyte is neurotoxic, which manifest itself as loss of homoeostatic functions responsible for synapse maintenance. In other study, selective astrocyte-specific targeting of unfolded protein response, which is exuberated in reactive astrocytes, was found to extend the incubation time to the terminal disease in mice [30]. This also suggests that reactive astrocytes are neurotoxic [30].

Among possible neuroprotective effects of the reactive state is phagocytosis of PrP^Sc^. Indeed, primary astrocyte cultures and astrocyte cell line were found capable of phagocytic uptake or internalization of PrP^Sc^ *in vitro* [31,32]. Moreover, PrP^Sc^ aggregates were found in reactive astrocytes in animals infected with prions [22,27,33]. However, whether phagocytic clearance is up- or downregulated in reactive state versus homoeostatic astrocytes has never been tested. It is also not clear whether reactive astrocytes can selectively upregulate phagocytosis of PrP^Sc^ but not synapses or viable neurons.

The net neurotoxic phenotype does not exclude upregulation of neuroprotective functions in parallel with the loss of homoeostatic functions and/or to activation of neurotoxic mechanisms (Figure 2). Moreover, considering significant region-specific heterogeneity in astrocyte homoeostatic phenotypes [4,9,34–36], asynchronous progression of the disease in different brain regions along with selective tropism of prion strains to different regions [37,38], it cannot be excluded that distinct reactive states with opposite net effects on neuronal survival are present in different brain regions during diseases time course.

Recent studies on transcriptome analysis of astrocyte-specific genes revealed that the manifestation of the reactive states associated with prion diseases was not in dysfunction of any specific pathway, but a global transformation of the physiological state of astrocytes, characterized by disturbance in multiple functions [26]. While both sets of genes, those involved in neuroprotection and neurotoxic functions, were disturbed, the net result of disturbances produced a neurotoxic phenotype [26]. Remarkably, the degree of astrocyte activation along with disturbance in functional pathways showed strong reverse correlation with the incubation time to disease [26]. The most rapid disease progression was found in the animal groups with the most severe astrocyte response. Analysis of astrocyte-specific genes raised the possibility that the degree of astrocyte activation contribute to the faster progression of the disease and perhaps even drive prion pathogenesis [26].

## Does microglia trigger astrocyte activation?

Because microglia and astrocytes often become reactive in parallel and both contribute to neuroinflammation, it is difficult to pinpoint the contribution of each cell type. According to the Barres hypothesis that gained popularity in neurodegeneration field, reactive microglia drives the polarization of astrocytes into neurotoxic A1 state via the upregulation of three secreted factors TNF-α, IL-1α and C1qa [39]. In conflict with this hypothesis, the ablation of these three factors in triple TNF^−/-^/IL1a^−/-^/C1qa^−/-^ knockout mice was found to accelerate the progression of prion diseases resulting in only modest suppression in A1-specific markers in prion-infected animals [23]. Moreover, contrary to this hypothesis, the partial ablation of microglia by PLX5622 exacerbated the reactive phenotype of astrocyte and, again, accelerated disease progression [40]. These studies argued that in prion diseases, instead of driving neurotoxic phenotype in astrocytes, reactive microglia seem to attenuate it. Moreover, it appears that the reactive phenotype of astrocyte is not dictated entirely by reactive microglia, and independent mechanisms of activation exist. Indeed, the analysis of transcriptome revealed that astrocytes respond to prion infection prior to clinical signs and, perhaps, even prior to microglia [21]. Astrocytes are sensitive to neuronal activity and respond to abnormalities in synaptic transmission [41]. Furthermore, studies that employed cell cultures showed that astrocytes can detect PrP^Sc^ and, in response, upregulate chemokine gene expression releasing signals that trigger microglia migration [42]. Reactive astrocytes isolated from prion-infected mice upregulate the expression of pro-inflammatory genes (*IL6, IL12b, IL33, Ccl4*) along with the secretion of IL-6, suggesting that the reactive phenotype is relatively stable and can be maintained *in vitro* in the absence of pro-inflammatory CNS environment [29]. IL-33 secreted by astrocytes is known to drive microglia-dependent synapse engulfment and elimination [43], while elevated levels of IL-6 triggers pathways are linked to neurodegeneration [44]. In mouse models of Alzheimer’s and Huntington’s disease, suppressing astrocyte reactivity by inhibiting activation of transcription factor STAT3 selectively in astrocytes reduced neuroinflammation and activation of microglia [45]. To summarize, the reactivity phenotypes of microglia and astrocytes appear to be closely coordinated and rely on multiple feedback loops. Both cell types are capable of serving as the first responders to CNS insults and drive neuroinflammation.

## Is reactive state of astrocyte reversable?

Is presence of a stimulus that triggers reactive states important for maintaining the reactive phenotype? Studies of optic nerve subjected to mild injury introduced by a brief ocular pressure showed that astrocyte reactivity could be fully resolved if the insult is removed [46]. In more severe insults such as spinal cord injury that lead to the formation of glial scares consisting of reactive astrocytes, phenotypic changes have long been considered irreversible. However, recent studies showed that reactive astrocytes isolated from injured spinal cord reverted their phenotype upon transplantation into a naïve spinal cord and vice versa [47], suggesting that preservation of reactive phenotypes relies on the persistent stimulus or the presence of environmental factors. In our study on isolation of astrocytes from prion-infected animals, the reactive phenotypes were preserved, at least in part, for three weeks post-isolation [29].

Is it possible to reverse reactive states in the presence of persistent stimulus? Activation of STAT3 transcription factor was identified as universal feature of astrocyte reactivity in neurodegenerative diseases shared between different species, brain regions and different types of illnesses (reviewed in [6,48]. Selective inhibition of STAT3 pathway in astrocytes was found to suppress astrocyte activation or reverse their reactive phenotype, and improved the disease outcomes in several animal models of neurodegenerative diseases [45,49,50]. Activation of STAT3 was observed in animals infected with prions [51]; however, its role in driving astrocyte reactive states associated with prion diseases has not yet been examined.

## What stimuli trigger astrocyte activation?

In prion diseases, astrocytes respond to prion infection prior to clinical symptoms or neuronal damage [21]. In mice infected with prions, the onset of GFAP upregulation appeared to be triggered by the accumulation of PrP^Sc^ over a certain threshold, while the kinetics in GFAP overexpression followed very closely the kinetics of PrP^Sc^ accumulation [52]. Phagocytosis of PrP^Sc^ *in vitro* by cultured astrocytes [31,32] along with intracellular localization of PrP^Sc^ aggregates in reactive astrocytes in animals [22,27,53] suggests that astrocytes have the ability to recognize PrP^Sc^ directly. Indeed, in response to scrapie brain homogenate, cultured astrocytes upregulated the expression of cytokine genes [42]. It is not clear what receptors are responsible for recognition of PrP^Sc ^ in astrocytes and neurons share the expression of lipoprotein receptor-related protein 1 (LR1P), which in neurons was found to be involved in endocytosis of PrP^Sc^ [54]. Astrocytes and microglia share a wide range or receptors that might participate in PrP^Sc^ phagocytosis, including toll-like receptors (TLRs), which are involved in the recognition of pathogens and extracellular protein aggregates; Axl receptor activates JAK/STAT pathway; a heterogeneous family of scavenger receptors is capable of recognition of danger- and pathogen-associated molecular patterns along with extracellular protein aggregates [55]; and MEGF10, an important receptor, is involved in phagocytosis on synapses and apoptotic cells [56]. Cultured microglia react to purified PrP^Sc^ by upregulating proinflammatory signalling [57]. Notably, the degree of response in microglia was found to be dictated by the sialylation status of N-linked glycans on the surface of PrP^Sc^ [57], with a stronger response caused by a PrP^Sc^ with desialylated glycans [57]. Like normal form of the prion protein or PrP^C^, PrP^Sc^ is sialylated [58]; however, the level and density of sialylation of PrP^Sc^ particles are variable among prion strains and dictated by a strain identity [59–62]. It is not known whether the same signalling pathways that respond in microglia to asialoglycans are also active in astrocytes. Remarkably, significant reduction in PrP^Sc^ sialylation levels that accompanied cross-species adaptation of a strain to a new host produced a new strain characterized by a very profound neuroinflammation and the shortest incubation time to the diseases [22]. These results suggested that a causative link between sialylation status of PrP^Sc^, the degree of glia activation and the rate of disease progression exists [22].

## Concluding remarks

With the development of new tools and gaining more knowledge regarding the role of reactive astrocyte in neurodegenerative disease, new questions have to be answered. To what extent the heterogeneity in homoeostatic state dictates reactive state of astrocytes? Do reactive phenotypes drive diseases pathogenesis? Is it possible to fully and selectively reverse reactive states of astrocytes in the presence of persistent proinflammatory stimulus? Does reversing of the reactive states represent an effective therapeutic approach? Answering these question brings a new opportunity for developing unexplored therapeutic approaches.

## Figures and Tables

**Table:** 

PrP^C^	normal cellular isoform of the prion protein
PrP^Sc^	abnormal, disease-associated isoform of the prion protein

## Abbreviations

PrP^C^, normal cellular isoform of the prion protein; PrP^Sc^, abnormal, disease-associated isoform of the prion protein
